# Non-Structural Protein 3 of Duck Tembusu Virus Induces Autophagy *via* the ERK and PI3K–AKT–mTOR Signaling Pathways

**DOI:** 10.3389/fimmu.2022.746890

**Published:** 2022-02-03

**Authors:** Jun Zhao, Tingting Zhang, Guomin Chen, Ningwei Geng, Zhiyun Guo, Shengliang Cao, Yudong Yang, Kuihao Liu, Siqi Wang, Yiran Zhao, Fanliang Meng, Sidang Liu, Meijie Jiang, Ning Li

**Affiliations:** ^1^ Shandong Provincial Key Laboratory of Animal Biotechnology and Disease Control and Prevention, Shandong Provincial Engineering Technology Research Center of Animal Disease Control and Prevention, College of Animal Science and Technology, Shandong Agricultural University, Taian City, China; ^2^ Collaborative Innovation Center for the Origin and Control of Emerging Infectious Diseases, College of Basic Medical Sciences, Shandong First Medical University, Taian City, China; ^3^ Laboratory Medicine, Central Hospital Affiliated to Shandong First Medical University, Jinan, China; ^4^ Laboratory Medicine, Tai’an City Central Hospital, Taian, China; ^5^ Sino-German Cooperative Research Centre for Zoonosis of Animal Origin Shandong Province, Shandong Agricultural University, Taian City, China

**Keywords:** duck Tembusu virus, non-structural protein 3, autophagy, ERK2, PI3K-AKT-mTOR, virus replication

## Abstract

Despite autophagy’s pivotal role in the replication of viruses such as duck Tembusu virus (DTMUV), which has caused massive economic losses to the poultry industry in the world, the specific relationships between DTMUV and cellular autophagy remain largely unknown. In response, we investigated the interactions between autophagy and DTMUV, the effects of the structural and non-structural proteins of DTMUV on autophagy, and the autophagy-related signaling pathways induced by DTMUV. Among the results, DTMUV increased the autophagy flux in duck embryo fibroblasts (DEF) and BHK-21 cells, while autophagy facilitated viral replication. After we pharmacologically induced autophagy with rapamycin (RAPA), the replication of DTMUV increased by 15.23-fold compared with the control group of DEF cells. To identify which DTMUV protein primarily induced autophagy, all three structural proteins and seven non-structural proteins of DTMUV were transfected into cells, and the results showed that non-structural protein 3 (NS3) induced significant autophagy in DEF cells. By means of Western blot, immunofluorescence, and transmission electron microscopy, we confirmed that NS3 protein could significantly induce autophagy and autophagy flux. Furthermore, we showed that NS3 induced autophagy in DEF cells through extracellular signal-regulated kinase 2 (ERK2) and phosphatidylinositol-3-kinase (PI3K)/AKT and the mammalian target of rapamycin (mTOR) signaling pathways using specific inhibitors and RNA interference assays. Finally, autophagy induced by NS3 promoted DTMUV replication. These results provide novel insight into the relationship between DTMUV and autophagy, broadening the current understanding of the molecular pathogenesis of DTMUV.

## Introduction

In April 2010, duck Tembusu virus (DTMUV) was identified as the causative agent of egg drop syndrome, a condition among egg-laying and breeder ducks in China characterized by depression, a substantial decrease in egg laying and growth, and signs of neurological degradation (1). Since then, expanding host profiles of DTMUV infection have revealed that it can infect not only ducks but chickens and geese as well (2). Even BALB/c mice infected with DTMUV have shown serious systemic and neurological symptoms (3). Moreover, when positive DTMUV antibodies were detected in the sera of workers at duck farms (4), the virus became known not only for causing tremendous economic losses in the poultry industry but also as a potential zoonotic pathogen that could threaten public health (5).

DTMUV is a single-stranded RNA virus belonging to the *Flavivirus* genus of the Flaviviridae family; this genus also includes many mosquito-borne flaviviruses, such as dengue virus (DENV), West Nile virus (WNV), Zika virus (ZIKV), and Japanese encephalitis virus (JEV) (6). DTMUV possesses a genome approximately 11 kb long that encodes three structural proteins (C, prM, and E) and seven non-structural proteins (NS1, NS2A, NS2B, NS3, NS4A, NS4B, and NS5) (7). Whereas the structural proteins primarily mediate the virus’ life cycle and fusion in the formation of viral particles (8, 9), the non-structural ones engage in viral replication, virion assembly, and innate immune evasion (10). NS3 is a multifunctional protein with three different activities, which are determined by serine protease, RNA helicase, and triphosphatase, respectively (11). NS3 also plays a non-enzymatic role in autophagy, fatty acid biosynthesis, and actin polymerization, all of which are mediated by its recruitment of host proteins from various cellular pathways (12).

Autophagy represents an intracellular catabolic pathway highly conserved among eukaryotes that entails the removal of long-lived, aggregated, unfolded proteins and damaged organelles, as well as the regulation of growth and senescence (13). Autophagy is monitored by the lipidation rate of microtubule-associated protein 1 light chain 3 (LC3) and the degradation of polyubiquitin-binding protein sequestosome 1 (SQSTM1/p62, and p62 hereafter) (14). When autophagy occurs, LC3 will be converted from LC3-I to LC3-II with phosphatidylethanolamine by E3-like conjugation enzymes, which are located on autophagosomes (15). Autophagy is regulated by many factors, including pathogenic microbial infections (16), hunger, and other adverse environmental factors (17). In addition, a variety of signal pathways are also involved in the regulation of autophagy. At present, there are many studies on adenosine 5′-monophosphate (AMP)-activated protein kinase (AMPK), extracellular signal-regulated kinase 2 (ERK2), AKT-mTOR, and so on (18).

Given the surprisingly close relationship between numerous viruses and autophagy (19), some viruses have evolved mechanisms to inhibit autophagy in infected cells in order to survive in hosts. In WNV, for instance, the capsid protein can inhibit autophagy *via* the AMPK pathway (20). Other flaviviruses, however, including DENV and JEV, have evolved mechanisms to benefit from autophagy (20). Recent studies have shown that DTMUV infection can induce autophagy *in vitro*, particularly by promoting viral replication by decreasing the phosphorylation of TANK-binding kinase 1 (TBK1) and inhibiting antiviral immune responses (21). *In vivo*, autophagy can also facilitate DTMUV replication and thus aggravate the development of pathological symptoms (22). Despite such knowledge, the specific molecular mechanism of autophagy triggered by DTMUV remains unclear. Because those structural and non-structural proteins of DTMUV play important roles in autophagy and viral replication (23), we screened for the viral protein that induces autophagy and studied the autophagy-related pathways that it mediates. Our results suggested that the non-structural protein NS3 can induce autophagy and thus affect viral replication through the ERK2 and PI3K-AKT-mTOR signaling pathways.

## Materials and Methods

### Ethics Statement

This study was carried out in accordance with the Guide for the Care and Use of Laboratory Animals of the Shandong Agricultural University.

### Cells, Virus, and Drugs

BHK-21 cells were obtained from the China Center for Type Culture Collection (Wuhan, China); the cell transfer algebra is within 10–20 generations. Duck embryo fibroblast (DEF) cells were isolated from 10-day-old duck embryos using trypsin digestion. Due to primary cell reasons, the transfer algebra is only within 1–3 generations. Both cells were cultured in Dulbecco’s modified Eagle’s medium (DMEM; Gibco, Langley, OK, USA) supplemented with 10% fetal bovine serum (Biological Industries, Beit HaEmek, Israel) at 37°C in 5% CO_2_ in a humidified incubator. The DTMUV FX2010 strain (accession: MH414568.1) proliferated on the BHK-21 cells, and the cellular supernatant was collected for subsequent assays. The titer of the FX2020 strain was determined on DEF cells *via* a 50% tissue culture infectious dose (TCID_50_) using the Reed–Muench method, in which the TCID_50_ of the stock virus was 10^5.4^ TCID_50_/100 µl. All drugs used in this study were purchased from MedChemExpress (MCE) company (Princeton, New Jersey,USA). When the cells were treated with 3-MA, cells were cultured in DMEM containing 3% FBS in a humidified incubator at 37°C with 5% CO_2_.

### Transmission Electron Microscopy

DEF cells grown to 70%–80% confluence in 6-well plates were infected with PBS or DTMUV FX2010 or transfected with pCMV-HA and pCMV-HA-NS3, respectively, and treated by RAPA as the positive control. The DEF cells of different treatments in each group were collected and centrifuged for 5 min at 800–1,000 r/min, then the supernatant was discarded, cell sediment at the bottom of the centrifuge tube was collected, and the buffer was added. After being centrifuged for 5 min at 5,000 r/min, the dense adherent cell pellet was collected. Then, we discard the supernatant and inject 2% to 4% glutaraldehyde (or a mixture of paraformaldehyde and glutaraldehyde), which was sent to Shandong Weiya Technology Co., Ltd., for transmission electron microscopy (TEM) observation. Images were obtained with a JSM-7500F transmission electron microscope (JEOL, Tokyo, Japan).

### Transfection and RNA Interference

All 10 proteins of DTMUV were cloned into the pCMV-HA vector in order to construct recombinant plasmids, then were subsequently transfected into DEF and BHK-21 cells using Lipofectamine 3000 (Invitrogen, Carlsbad, CA, USA) in accordance with the manufacturer’s instructions. Once the cells were collected at 24 h post-transfection (hpt) for Western blot analyses, DEF cells were grown in 6-well plates and transiently transfected with siRNAs (**Table 1**) using a transfection reagent Lipofectamine RNAiMAX (Invitrogen, Carlsbad, CA, USA), also in accordance with the manufacturer’s instructions. Knockdown efficiencies were determined by Western blot analysis. At 24 hpt, the cells were mock-infected with DMEM or inoculated with the FX2010 strain at a multiplicity of infection (MOI) of 0.3 and harvested at 36 h postinfection (hpi). The infected cells were harvested for the following assays.

**Table 1:** Sequences of siRNA for ERK2 and primers used for qPCR detection.

Types	Name	Sense (5′–3′)	Antisense (5′–3′)
siRNAs	ERK2 317	CACCCUUCAAGUUUGAUAUTT	AUAUCAAACUUGAAGGGUGTT
	ERK2 1023	GCCAGGAUAUCCAUCUUAATT	UUAAGAUGGAUAUCCUGGCTT
	ERK2 1108	GCUCCAACUAUUGAACAAATT	UUUGUUCAAUAGUUGGAGCTT
	Negative control	UUCUCCGAACGUGUCACGUTT	ACGUGACACGUUCGGAGAATT
qPCR primers	β-Actin	GAGAAATTGTGCGTGACATCA	CCTGAACCTCTCATTGCCA
	ATG5	CCTCCTGAAGCAATTGGTCG	AGCAACTGTGGATGGGGTTA

### mRFP-GFP-LC3 Transfection

BHK-21 cells were seeded onto glass coverslips in 24-well plates, and the mRFP-GFP-LC3 plasmid was subsequently transfected into cells using Lipofectamine 3000 (Invitrogen, Carlsbad, CA, USA) in accordance with the manufacturer’s instructions. After being transfected for 24 h, BHK-21 cells were transfected with NS3, infected with virus, or treated with other drugs for 24 h. A laser scanning confocal microscope (Leica, Wetzlar, Germany) was used to observe autophagy flux. Yellow (overlay of mRFP and GFP) fluorescence spots indicated early autophagosomes; red (mRFP alone) fluorescence spots indicated late autolysosomes.

### Indirect Immunofluorescence Assay

DEF cells were seeded on coverslips and co-transfected with pEGFP-LC3 and 10 recombinant plasmids as well as empty vectors, respectively. At 24 hpt, the cells were fixed with 4% formaldehyde and permeabilized with 0.1% (v/v) Triton X-100 in PBS. The transfected DEF cells were probed with rabbit-origin anti-HA monoclonal antibody (Abcam, Cambridge, UK), and the HA recombinant proteins were visualized using Cy3 goat anti-rabbit immunoglobulin (IgG; Jackson, West Grove, PA, USA). All probed cells were observed under a Leica SPE confocal microscope (Wetzlar, Germany).

### Real-Time Quantitative PCR

Total RNAs were extracted from cells using the GeneJET RNA Purification Kit (Thermo Scientific, Waltham, MA, USA) and reverse-transcribed using the ReverTra Ace qPCR RT Kit (Toyobo, Osaka, Japan) according to the manufacturers’ instructions. The specific primer of the DTMUV E gene and reaction conditions were reported previously (24), and the relative quantitative PCR (qPCR) was used to quantify the E gene expression with abundant β-actin mRNA as the internal control. All qPCR tests were performed using the SYBR Green Real-Time PCR Master Mix (Toyobo, Osaka, Japan) using a LightCycler 96 (Roche, Basel, Switzerland).

### Western Blot Analysis

Cellular proteins were separated by 8%–12% SDS-PAGE and transferred to polyvinylidene difluoride membranes (Millipore, Bedford, MA, USA) using a semidry transfer apparatus (Bio-Rad, Hercules, CA, USA) in accordance with standard procedures. The primary antibodies used for detecting viral and host proteins included the monoclonal antibody against DTMUV E protein, the HA antibody (3724S; Cell Signaling Technology, Boston, MA, USA), an anti-β-actin IgG (4970T; Cell Signaling Technology), anti-cell autophagy signal pathway proteins, and their phosphorylated protein antibodies (Cell Signaling Technology)—that is, PI3K (4249T), AKT (4691T and p-AKT 4060S), mTOR (2983T and p-mTOR 5536T), and ERK2 (4695T and pERK2 4370T). Autophagy marker proteins LC3 (L8918) and P62 (P0067) were purchased from Sigma (San Francisco, CA, USA). Monoclonal antibody E was obtained from mice, and other antibodies were rabbits. Horseradish peroxidase-conjugated anti-mouse and anti-rabbit antibodies (Jackson, Lancaster, PA, USA) were used as secondary antibodies. Protein bands were visualized using the Clarity Western ECL substrate (Bio-Rad, Hercules, CA, USA).

### Statistical Analysis

The data were recorded as the M ± SD of at least three independent experiments. SPSS version 25.0 (SPSS Inc., Chicago, IL, USA) was used for statistical analysis, and *p* value was calculated with a two-tailed paired or unpaired Student’s *t* test in GraphPad version 8.0 (Prism, San Diego, CA, USA). Values of *p < 0.*05 were considered to be statistically significant, *p < 0.05; **p < 0.01.

## Results

### The NS3 of DTMUV Induces Autophagy

**Figure 1:**
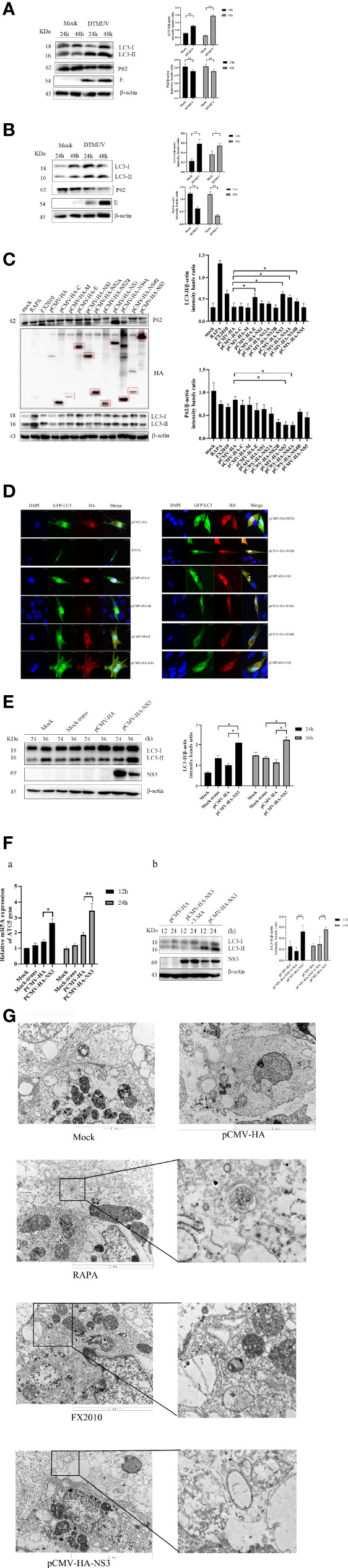
Autophagy in cells infected with DTMUV and transfected with NS3. **(A)** DEF cells and **(B)** BHK-21 cells were mock-infected or infected with DTMUV at an MOI of 0.3 for 24 and 48 h. Samples were harvested for Western blot analysis and immunoblotted for proteins LC3, E, P62, and β-actin. On the right is the statistics and analysis of the Western blotting data (the same below). **(C)** DEF cells were transfected with pCMV-HA empty vector and all 10 protein plasmids for 24 h. Samples were harvested for immunoblotting. **(D)** DEF cells were transfected with pEGFP-LC3 and each DTMUV protein plasmid as the experimental group, after which cells were transfected with pEGFP-LC3 and pCMV-HA plasmids as negative controls, and the RAPA-treated group (5 μM) was transfected with pEGFP-LC3 as a positive control. Cells were fixed and imaged for GFP fluorescence after transfection for 24 h. The images shown represent three independent experiments. **(E)** DEF cells were transfected with pCMV-HA and pCMV-HA-NS3 plasmids for 24 and 36 h while the mock group and mock transfection group were arranged. **(F)** a.) DEF cells were transfected with pCMV-HA and pCMV-HA-NS3 plasmids for 12 and 24 h while the mock group and mock transfection group were arranged. The total RNA of the cells was extracted, and the transcriptional level of ATG5 gene was detected by reverse transcription qPCR. b.) After 6 h of treatment with 3-MA (5 mM), the pCMV-HA-NS3 plasmid was transfected into DEF cells, and samples were collected at 12 and 24 hpt. Proteins levels of LC3 and β-actin were examined by Western blot. **(G)** DEF cells were transfected with pCMV-HA and pCMV-HA-NS3 plasmids for 24 h or treated by RAPA (5 μM) for 4 h as positive control. DEF cells were infected with MOI = 1 virus for 24 h. All the samples were collected and observed by electron microscope. The density of the protein bands measured with fusion analysis in ImageJ software was determined after subtracting the density of the β-actin bands. The β-actin protein was used as the internal control. Data (M ± SD of technical triplicates) represent three independent experiments or are pooled from three independent experiments (M ± SD). *p < 0.05; **p < *0.*01.

To monitor autophagy after DTMUV infection, we analyzed the change of autophagic marker protein LC3. As shown in **Figures 1A, B**, the expression of LC3-II significantly increased at 24 and 48 hpi in BHK-21 and DEF cells infected with DTMUV, whereas the amount of LC3-II in the mock group did not increase as significantly. The results of Western blot also demonstrated that the accumulation of LC3-II increased with viral replication, especially in the late stages of DEF cell infection. Statistical analysis showed that autophagy levels of the mock group and virus infection group were significantly different (*p <* 0.01).

To further identify which proteins of DTMUV induced autophagy, we individually cloned all 10 genes (C-NS5) of DTMUV into the pCMV-HA vector and transfected into DEF cells to detect protein expression and autophagy by Western blot. The results indicated that all proteins could be expressed (**Figure 1C**). Next, we detected the change in the LC3-II protein and found that the E, NS3, NS4A, and NS4B proteins could significantly increase its amount and decrease the expression of P62, thereby suggesting that they might induce autophagy in DEF cells (**Figure 1C**). To provide additional evidence, pEGFP-LC3 and viral protein plasmids were co-transfected DEF cells for 24 h. Afterward, confocal microscopy was used to analyze the punctate accumulation of the fluorescent LC3 and its co-localization between LC3 and viral proteins in the transfected cells. As shown in **Figure 1D**, a strong, green punctate accumulation of the pEGFP-LC3 protein in the cytosol was observed in the cells expressing NS3 protein, and NS3, marked as red, was entirely located in the cytoplasm in an aggregated manner, whereas the control GFP protein diffusively distributed throughout the cell transfected with pCMV-HA. There was a high degree of co-localization between NS3 and pEGFP-LC3 proteins. According to those results, we speculated that NS3 could induce autophagy in DEF cells.

To study the characteristics of autophagy induced by NS3, we observed the LC3-II protein at 24 and 36 hpt. As shown in **Figure 1E**, compared with the empty vector group, the content of LC3-II in cells transfected with pCMV-HA-NS3 increased significantly from 24 to 36 hpt (*p <* 0.05); moreover, the autophagy level induced by NS3 is significantly higher than those of mock-trans and pCMV-HA (**Figure 1E**). Further studies showed that NS3 protein could increase the transcription level of autophagy-related gene ATG5 (*p <* 0.05), and 3-MA could effectively inhibit NS3-induced autophagy in DEF cells 24 h after transfection (**Figure 1F**). Because the formation of autophagosomes more directly determines whether NS3 triggers autophagy, we first observed the formation of autophagosome-like vesicles in DEF cells by the TEM method. In this study, we use Rapa treatment in DEF cells as a positive control and found some autophagosome-like vesicles (**Figure 1G**) in Rapa-treated DEF cells; a similar phenomenon occurred in the virus-infected group, but just a few in mock cells. Interestingly, we also observed the fusion of autophagosomes and lysosomes in the group transfected with the NS3 plasmid. Those findings showed that DTMUV NS3 could induce autophagy in DEF cells.

### NS3 Can Cause Complete Autophagy Flux

**Figure 2:**
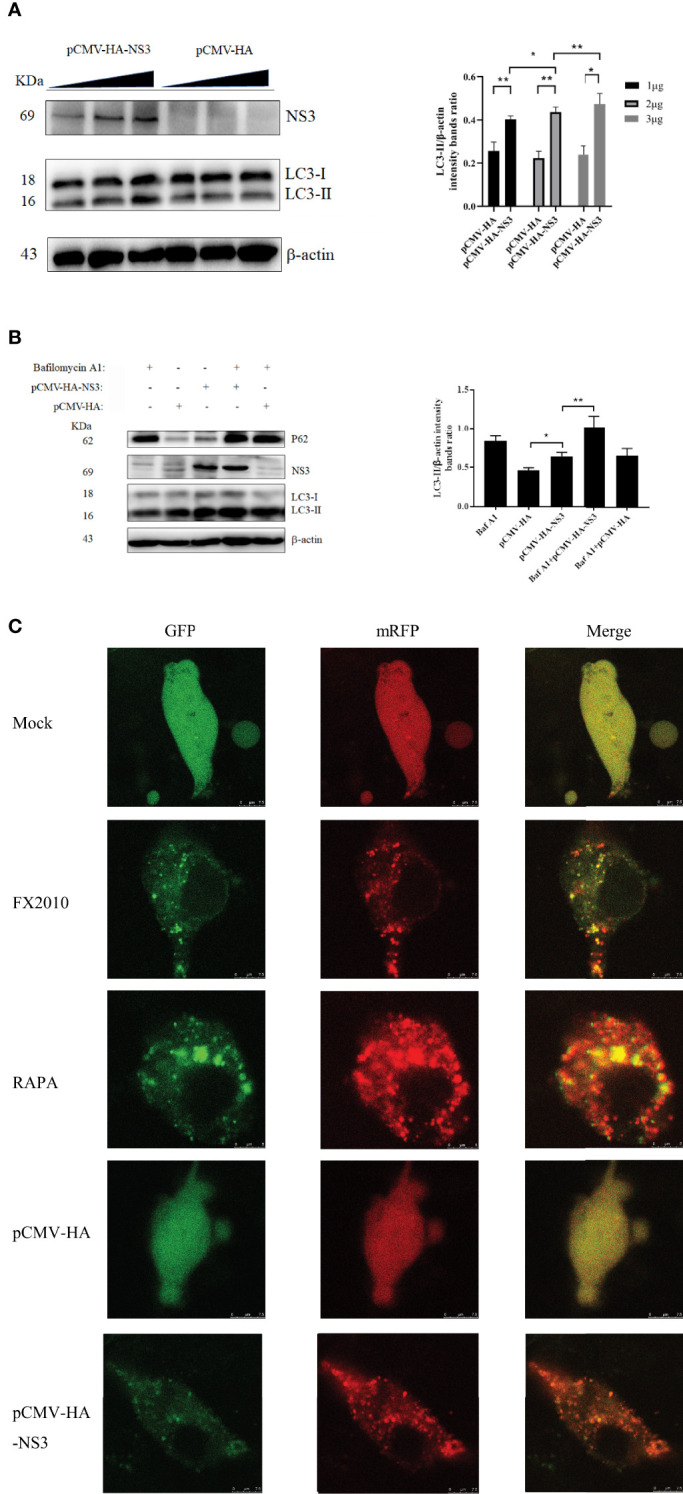
NS3 can cause complete autophagy flux. **(A)** The amount of NS3 or pCMV-HA plasmids transfected into each six-well plate cells was 1 mg, 2 mg, and 3 mg, respectively. Samples were harvested at 24 hpt for Western blot analysis. **(B)** After 24 h of treatment with Bafilomycin A1 (400 nM), the pCMV-HA and pCMV-HA-NS3 plasmids were transfected into DEF cells respectively, and samples were collected at 24 hpt. **(C)** DEF cells were transfected with mRFP-GFP-LC3 plasmids for 24 h. Then proceed to the following processing group. DEF cells were transfected with pCMV-HA and pCMV-HA-NS3 plasmids respectively or treated by RAPA (5 μM) for 4 h as positive control or infected with MOI = 1 virus for 24 h. All the samples were collected and observed by electron microscope. The density of the protein bands measured with fusion analysis in ImageJ software was determined after subtracting the density of the β-actin bands. The β-actin protein was used as the internal control. Data (M ± SD of technical triplicates) represent three independent experiments or are pooled from three independent experiments (M ± SD). *p < 0.05; **p < 0.01.

To further investigate whether autophagy induced by NS3 was dose-dependent, we transfected pCMV-HA-NS3 and empty vector plasmids with 1, 2, and 3 μg, respectively; the results revealed a significant dose-dependent relationship between autophagy and NS3 protein (**Figure 2A**). Previous results showed a decline in the P62 protein (**Figures 1A, B** **)**, which suggested that DTMUV can cause complete autophagy flux in cells. Bafilomycin A1 disrupts autophagic flux by inhibiting both V-ATPase-dependent acidification and Ca-P60A/SERCA-dependent autophagosome–lysosome fusion (25). Therefore, to further investigate whether NS3 can cause complete autophagy flux, DEF cells were treated with Bafilomycin A1 and then transfected NS3 protein. The results showed that after inhibition of autophagy flux, the continued expression and accumulation of LC3-II were caused by autophagy induced by the NS3 protein (**Figure 2B**). Statistical analysis showed that LC3-II levels of the NS3-transfection group and NS3 and Bafilomycin A1 co-treatment group were significantly different (*p <* 0.01). Moreover, we want to study the phenomenon of NS3-induced autophagy flux further and more intuitively, as shown in **Figure 2C**; after transfection with mRFP-GFP-LC3 in BHK-21 cells, we observed that the number of red fluorescence spots indicating late autolysosomes was significantly increased in the NS3 and FX2010 groups compared with the control group. However, the intensity of green fluorescence decreased, indicating that green fluorescence quenching was caused by an acidic environment when virus or NS3 induced autophagy. To sum up, NS3 can cause complete autophagy flux.

### NS3 Induces Autophagy by Promoting the Phosphorylation of ERK2

**Figure 3:**
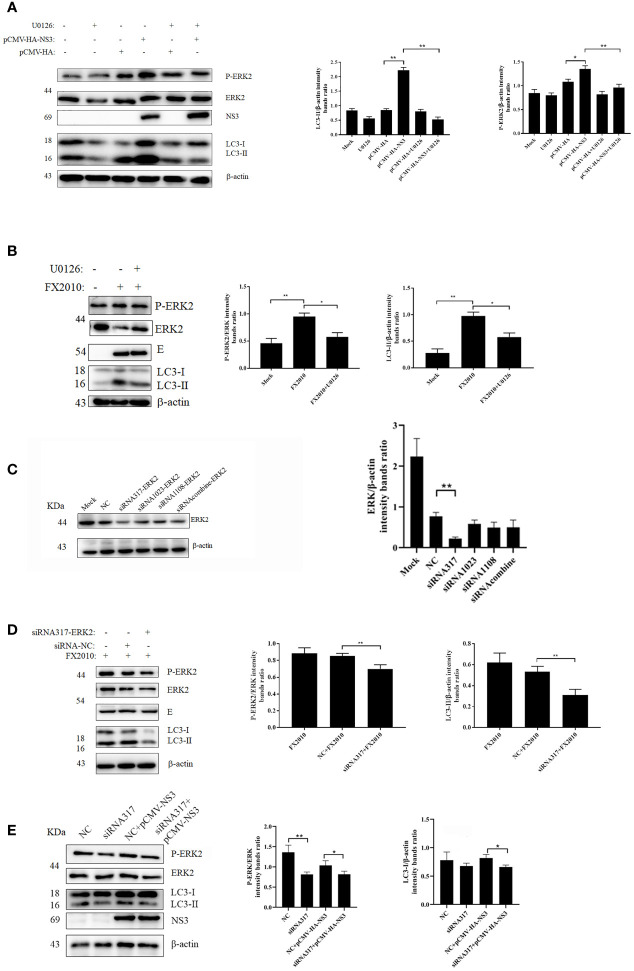
NS3-induced autophagy by promoting the phosphorylation of ERK. **(A)** After 2 h of treatment with U0126 (20 μM), the pCMV-HA and pCMV-HA-NS3 plasmid was transfected into DEF cells respectively, and samples were collected at 24 hpt. **(B)** DEF cells infected with MOI = 0.1 virus for 24 h. **(C)** DEF cells were transfected with three siRNAs for ERK2 and negative control siRNA for 24 h, after which the samples were used for Western blot analysis. **(D)** After the transfection of siRNA or NC into DEF cells for 24 h, cells were infected with MOI = 0.1 virus. After 24 h, the samples were collected for autophagy analysis. **(E)** After the transfection of siRNA or negative control into DEF cells for 24 h, the pCMV-HA-NS3 plasmid was transfected. After 24 h, the samples were collected for autophagy analysis. The density of the protein bands measured with fusion analysis in ImageJ software was determined after subtracting the density of the β-actin bands. The β-actin protein was used as the internal control. Data (M ± SD of technical triplicates) represent three independent experiments or are pooled from three independent experiments (M ± SD). *p < 0.05; **p < 0.01.

Several studies have demonstrated that autophagy is associated with the ERK1/2 signaling pathway (26). ERK2 is a homologous analogue of ERK1/2 in duck, so we mainly detected the changes of ERK2 in DEF cells infected with DTMUV and transfected with the NS3 plasmid. We found that infection with virus or transfection with NS3 significantly increased the phosphorylation of ERK2 (Thr202 and Tyr204) (*p <* 0.01), accompanied by the accumulation of the LC3-II protein (**Figures 3A, B** **)**. U0126 could inhibit the phosphorylation level of ERK, which can treat the increase of LC3-II induced by virus (**Figure 3B**) or NS3. These results suggest that ERK2 is involved in NS3-induced autophagy. Furthermore, we designed siRNAs for ERK2 and verified their effects, which revealed that the interference effect of siRNA317 was the most significant difference (*p <* 0.01) (**Figure 3C**). DEF cells were pretreated with siRNA317 for 24 h and then infected with virus or transfected with NS3 protein. The levels of intracellular autophagy and ERK2 phosphorylation were measured. The results showed that inhibition of ERK2 phosphorylation could reduce the level of virus-induced autophagy (**Figure 3D**). In the same way, DEF cells were treated with siRNA317 prior to NS3 transfection. The results revealed that NS3-induced autophagy was repressed in the pretreated cells with siRNA317 (**Figure 3E**). To sum up, NS3 induced autophagy by promoting the phosphorylation of ERK2.

### NS3 Induces Autophagy by Inhibiting the Phosphorylation of PI3K–AKT–mTOR

**Figure 4:**
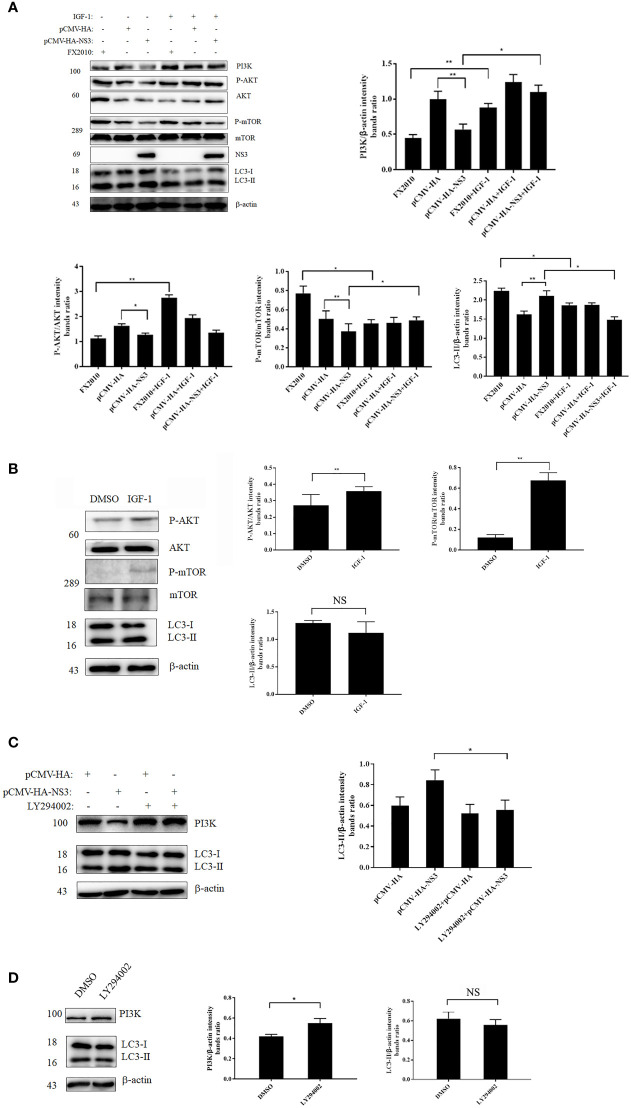
NS3 induces autophagy by inhibiting the phosphorylation of PI3K-AKT-mTOR. **(A)** After 24 h of treatment with IGF-1, the pCMV-HA and pCMV-HA-NS3 plasmids were transfected into DEF cells respectively, and samples were collected at 24 hpt. In addition, DEF cells were infected by FX2010 (MOI = 0.1) and samples were collected at 24 hpi. **(C)** After 2 h of treatment with LY294002 (10 μM), the pCMV-HA and pCMV-HA-NS3 plasmids were transfected into DEF cells respectively, and samples were collected at 24 hpt. **(B, D)** After 24 h of treatment with DMSO or IGF-1 (diluted by DMSO; 50 μg/ml), or treatment with LY294002 for 2 h, samples were collected at the corresponding time. Proteins levels of PI3K, AKT, mTOR, and their phosphorylated proteins, as well as β-actin, were measured by Western blot. The density of the protein bands measured with fusion analysis in ImageJ software was determined after subtracting the density of the β-actin bands. The β-actin protein was used as the internal control. Data (M ± SD of technical triplicates) represent three independent experiments or are pooled from three independent experiments (M ± SD). *p < 0.05; **p < 0.01; NS, no significance.

In our study, we found that NS3 protein inhibited the phosphorylation of AKT and mTOR compared with the empty vector, and the expression of PI3K decreased in the NS3 transfection group (**Figure 4A**). To further determine the role of this signaling pathway in NS3-induced autophagy, IGF-1, a physiology activator of AKT/mTOR, was selected for treatment, and the function of IGF-1 in DEF cells had been verified, as shown in **Figure 4B**; it was found that IGF-1 could significantly reduce the increase of autophagy induced by NS3 protein and DTMUV (**Figure 4A**). To further determine the role of PI3K in NS3-induced autophagy, we treated DEF cells with LY294002, which can regulate autophagy *via* PI3K, before transfecting NS3. As shown in **Figure 4C**, the effect of LY294002 on PI3K expression was also verified in DEF cells. Treatment with LY294002 resulted in decreased ratios of LC3-II in NS3-transfected cells compared with the group transfected with NS3 recombinant plasmid alone, thereby demonstrating that LY294002 effectively inhibits autophagy induced by NS3 (**Figure 4D**). These results suggest that PI3K is involved in NS3-induced autophagy. To sum up, these results further demonstrated that DTMUV NS3 protein induced autophagy *via* the PI3K–AKT–mTOR pathway in DEF cells.

### Autophagy Induced by NS3 Protein Promotes the Replication of DTMUV

**Figure 5:**
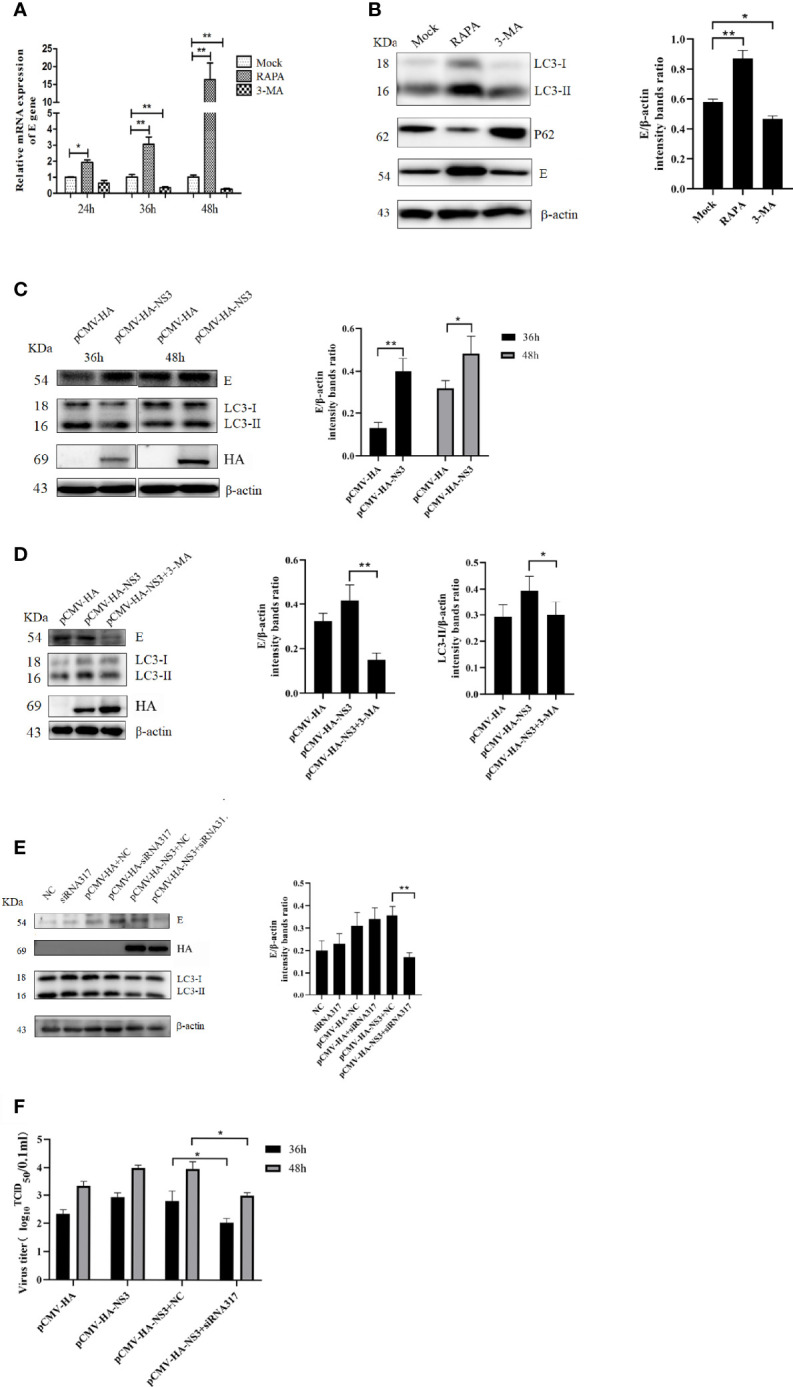
Autophagy affects DTMUV’s replication. **(A)** DEF cells were treated with 3-MA (5 mM) or RAPA (5 μM) for 6 h to inhibit or induce autophagy, respectively, before being infected with DTMUV at an MOI of 0.3. Cell lysates were harvested at the indicated time for qPCR to test the relative expression of the E gene. **(B)** DEF cells infected with DTMUV for 36 h were lysed and analyzed by Western blot with anti-LC3, P62, and the E and β-actin antibodies. **(C)** BHK-21 cells were transfected with NS3 for 6 h and then infected with DTMUV (0.3 MOI), and the cell samples were collected at 36 and 48 hpi. **(D)** BHK-21 cells were treated with 3-MA (5 mM) for 6 h before being transfected with NS3 and subsequently infected with DTMUV. At 36 hpi, the E, LC3, and NS3 proteins were detected by Western blot. **(E)** DEF cells were transfected with NS3 for 6 h after being transfected siRNA for 24 h. The cells were infected with DTMUV (0.3 MOI), and the cell samples were collected at 36 hpi. **(F)** DEF cells were cultured in 6-well cell culture plates and infected with the virus (0.3 MOI) as described above. Cell supernatants were collected 36 and 48 h after infection, and virus titers were determined by the Reed–Muench two-step method. Data (M ± SD of technical triplicates) represent three independent experiments or are pooled from three independent experiments (M ± SD). *p < 0.05; **p < 0.01.

To determine the effect of autophagy on the replication of DTMUV, we treated DEF cells with 3-MA or RAPA before DTMUV infection with the same MOI of 1. Once total RNA was extracted at 24, 36, and 48 hpi, the results of qPCR were analyzed. As shown in **Figure 5A**, at 24 hpi the relative expression of the E gene in the RAPA-treated group was twice as high as that in the mock group and the 3-MA-treated group. At 36 hpi, the expression of the E gene in the RAPA-treated group was 3.2-fold and 10.3-fold higher than that in the mock and 3-MA groups, respectively. As for infection with DTMUV after 48 h, the virus replicated rapidly, and the difference between the RAPA-treated group and the other two groups widened (*p <* 0.01). In addition, protein samples at 36 h were also collected to detect the expression of E protein; the results showed that the E protein in the RAPA-treated group was significantly higher than that in the other two groups. Consistently, 3-MA inhibited the replication of DTMUV (**Figure 5B**) (*p <* 0.01).

To determine whether autophagy induced by DTMUV NS3 also promotes viral replication, the groups were set up, as shown in **Figure 5C**, and the Western blot results showed that the overexpression of NS3 significantly promoted virus replication at 36 and 48 hpi compared with the empty vector group (*p <* 0.05). However, when autophagy induced by NS3 was inhibited by 3-MA or siRNA317, the ability of NS3 to promote replication was strongly inhibited (**Figures 5D, E**). At the same time, we measured the viral titer, and the results showed that inhibition of NS3-induced autophagy could reduce (*p <* 0.05) the viral titer of DTMUV (**Figure 5F**). Those data suggest that autophagy induced by NS3 promotes the replication of DTMUV.

## Discussion

Autophagy is a catabolic pathway used by eukaryotic cells to counteract stress response, promote organelle turnover, and clear aggregated proteins and excess lipids (27), as well as degrade infectious microorganisms and participate in the regulation of immune responses (28). In those ways, autophagy protects cells from damage and maintains cellular homeostasis (29). A complex relationship exists among autophagy, viruses, and their hosts. In this study, we first verified the interaction between DTMUV and autophagy; the results are consistent with the previous report (21). DTMUV infection can induce autophagy, and autophagy can promote virus replication. More importantly, we further studied the molecular mechanism of autophagy induced by DTMUV. Flavivirus encodes 10 proteins, of which 7 non-structural proteins play important roles in the interactions between virus and host. Studies have shown that the NS4A of a variety of flaviviruses plays a vital role in inducing autophagy, such as DENV (30), WNV (31), and ZIKV (32). When expressed alone, NS4A can significantly induce autophagy and affect the replication and pathogenicity of these viruses. However, the C, M, and NS3 proteins of JEV are also reported as the main proteins that induce autophagy in BHK-21 cells (33). The NS4B protein of hepatitis C virus (HCV) was sufficient to induce the autophagic vacuoles (34). Moreover, the NS3/4A complex and the NS5 of HCV individually were also able to induce autophagy (35). In the current study, further expression of individual DTMUV proteins indicated that E, NS3, NS4A, and NS4B proteins could significantly increase the expression of LC3-II, and we further confirmed that DTMUV NS3 protein played a key role in inducing autophagy of DEF cells by immunofluorescence, Western blot, and TEM experiments, and NS3 had the ability to induce complete autophagic flux in DEF cells. NS3 ranks among the best-characterized non-structural proteins of flaviviruses, which is a multifunctional protein with serine protease, RNA helicase, and triphosphatase activities (36, 37). Because the NS2B protein of flavivirus forms a stable complex with NS3 and acts as a cofactor for the NS2B-NS3 serine protease (38), we speculated that autophagy induced by NS3 has nothing to do with hydrolase, although the details warrant further examination. Interestingly, both the NS4A and NS4B proteins of DTMUV can also increase the amount of LC3-II. Considering the key roles of NS4A, 4B in other flavivirus-induced autophagy, especially NS4A, it is necessary to further verify the effect of DTMUV NS4A on autophagy.

Autophagy is a complex physiological metabolic process, which is affected by many factors, for example, PI3K–AKT–mTOR and the mitogen-activated protein (MAP) kinase ERK1/2 (39). In order to further clarify the signal pathway of autophagy induced by NS3 of DTMUV, inhibitors LY294002 and IGF-1-targeted PI3K–AKT–mTOR signal were used, then they decreased the autophagy induced by NS3, indicating that DTMUV NS3 protein could induce the autophagy of DEF cells through the PI3K–AKT–mTOR signaling pathway. PI3K–AKT–mTOR is a classic pathway for flaviviruses to regulate autophagy, and it has been reported that DENV NS4A can activate autophagy in a PI3K-dependent manner (30). Interestingly, ZIKV NS4A and NS4B have been shown to induce the autophagy of human fetal neural stem cells by deregulating the AKT–mTOR signal pathway (40), and HCV could also induce endoplasmic reticulum stress (ERS)-mediated unfolded protein response and inhibit the AKT–mTOR pathway to trigger autophagy (41). Besides, it has been reported that HCV NS3 might interact with ATG5 and ATG10 *via* immunity-related GTPase family M protein (IRGM) to induce autophagy, and IRGM is critical for HCV-induced autophagy, as its depletion suppressed HCV-induced autophagy (42). In fact, we also identified the host proteins that may interact with DTMUV NS3 by mass spectrometry and did not find IRGM and autophagy-related proteins but identified a variety of other proteins, such as heat shock protein (HSP) 90α, HSP90β, and HSP70. The specific interactions between them and NS3 and their roles in NS3-induced autophagy will be focused in our further study.

Recent studies have shown that infectious bronchitis virus can induce autophagy by ERK1/2 and play a pro-survival role at the late stage of IBV infection (43). Autophagy can be induced through ERK1/2 in PCV2-infected cells (44). JEV infects mouse microglia resulting in the activation of ERK/MAPKp38/AP-1/NF-κB signaling cascades, leading to the production of inflammatory cytokines (45), but it is not known whether ERK1/2 is related to autophagy induced by JEV. In this study, we found that DTMUV and NS3 protein can induce the phosphorylation of ERK2 in DEF cells, and the use of U0126 and interference RNA-targeted ERK2 can significantly inhibit DTMUV- and NS3-induced autophagy, indicating that ERK2 was required for the NS3-induced autophagy. Moreover, ERK2 regulated inactivated Sendai virus and induced autophagy in HeLa cells by inhibiting the PI3K–AKT–mTOR pathway (46). However, the relationships between the PI3K–AKT–mTOR and ERK2 signaling pathways in our study require further study.

The relationship between virus replication and autophagy is complicated, and the propagation of some viruses is suppressed by autophagy, whereas other viruses can hijack autophagy to promote their replication (47), such as autophagy which positively regulates JEV replication by regulating the early step of virus replication in neuronal cells (48), and autophagy activation has been found to increase the titer of DENV and enhance its pathogenicity in mice (49, 50), but it has also been reported that autophagy induced by WNV inhibits virus replication (36). Our results are consistent with previous reports that DTMUV-induced autophagy can enhance self-replication *in vivo* and *in vitro* (22), and NS3-induced autophagy can indeed promote virus replication. The possible mechanism was that DTMUV inhibited TBK-1 phosphorylation through P62 degradation in DEF cells, which led to the decrease of NF-κB and IRF3 phosphorylation and ultimately inhibited the expression of IFN-I and IL-6 (21).

In sum, the NS3 protein of DTMUV can induce autophagy in DEF cells *via* the ERK2 and PI3K–AKT–mTOR signaling pathways, while autophagy induced by NS3 can promote viral replication. Those results illuminate the pathogenesis of infection with DTMUV and provide novel insights into the development of effective therapeutic strategies.

## Data Availability Statement

The original contributions presented in the study are included in the article/supplementary material. Further inquiries can be directed to the corresponding authors.

## Author Contributions

JZ performed the most experiments, analyzed data and wrote the manuscript. TZ and GC made great efforts to revise the manuscript, NG, ZG, SC and YY helped construct the pCMV-HA-FX2010 plasmids. KL, SW, YZ and FM performed some western blot experiments. SL and MJ designed some experiments, NL designed the whole study and reviewed the article. All authors contributed to the article and approved the submitted version.

## Funding

This research was supported by the Natural Science Foundation of Shandong Province (ZR2021QC004); National Key Research and Development Program (Grants No. 2016YFD0500106); China Postdoctoral Science Foundation Funded Project (Grants No. 2019M652450); High-level Scientific Research Foundation for the introduction of talent of Shandong Agricultural University; and Funds of Shandong “Double Tops” Progra5m.

## Conflict of Interest

The authors declare that the research was conducted in the absence of any commercial or financial relationships that could be construed as a potential conflict of interest.

## Publisher’s Note

All claims expressed in this article are solely those of the authors and do not necessarily represent those of their affiliated organizations, or those of the publisher, the editors and the reviewers. Any product that may be evaluated in this article, or claim that may be made by its manufacturer, is not guaranteed or endorsed by the publisher.
